# Commentary: The Effect of Repetitive Transcranial Magnetic Stimulation on Dysphagia After Stroke: A Systematic Review and Meta-Analysis

**DOI:** 10.3389/fnins.2022.832280

**Published:** 2022-03-23

**Authors:** Yu-lei Xie, Shan Wang, Yu-han Xie, Xin Chen, Yin-xu Wang, Qing Wu

**Affiliations:** ^1^Department of Rehabilitation Medicine, Affiliated Hospital of North Sichuan Medical College, Nanchong, China; ^2^North Sichuan Medical College, Nanchong, China; ^3^University of South China, Hengyang, China

**Keywords:** deglutition disorders, transcranial magnetic stimulation, stroke, meta-analysis, commentary

About 29–81% of post-stroke survivors suffer from dysphagia, which is characterized by varying degrees of eating disorders, choking cough, salivation, and abnormal pronunciation. Dysphagia is associated with increased risk of malnutrition and pneumonia and leads to prolonged hospital stay, poor prognosis, and mortality (Park et al., 2017; Pandian et al., 2018; Alamer et al., 2020). However, the effect of repetitive transcranial magnetic stimulation (rTMS) on post-stroke dysphagia is not clear (Lefaucheur et al., 2020).

Therefore, the article of Yang et al. (2021) is very timely. Nevertheless, in this review, we have two main issues that question the validity of their results: (1) missing studies and (2) incorrect data extraction/analysis.

First, although Yang et al. searched relevant databases, they missed several randomized controlled trials that met the inclusion criteria in their review, such as Du et al. (2016), Cheng et al. (2017), and Tarameshlu et al. (2019), which were indexed in PubMed and Web of Science. This suggests that Yang et al. may not have strictly followed their narrative that “two evaluators (Weiwei Yang and Xiaoyun Zhang) independently assessed eligibility for inclusion in the analysis and extracted the relevant material according to predefined inclusion and exclusion criteria.”

Meanwhile, Yang et al. did not specify outcome indicators in the inclusion and exclusion criteria section, but only included studies involving the Permeation-Aspiration Scale (PAS) and Dysphagia Grade (DD) in the meta-analysis. The reasons are unclear. DD is mainly assessed using questionnaires, whereas PAS requires the collection of video images for assessment by the Fiber Endoscopic Evaluation of Swallowing (FEES) or the Video Fluoroscopic Swallowing Study (VFSS). The Standardized Swallowing Assessment (SSA), Functional Dysphagia Scale (FDS), and Mann Assessment of Swallowing Ability (MASA) are commonly used as measurements of swallowing function, and some trials using these outcome indicators have been missed. Moreover, a trial (Du et al., 2016) using DD was also not included in the meta-analysis. Yang et al. noted that this trial had a small sample size and was not a randomized controlled trial. The fact is this randomized controlled trial applied both DD and SSA to assess swallowing function and had an adequate sample size (*n* = 40). For PAS, some studies used both liquid and semi-solid measurements and further produced two different PAS results (Lim et al., 2014; Unluer et al., 2019), while other studies included thick liquid, semi-solid, and thin liquid measurements and chose the average value as the final result (Park et al., 2017). Therefore, Yang et al. should clearly define what type of PAS results could be included. In addition, some studies (Lim et al., 2014; Unluer et al., 2019) included both FDS and PAS, so it may have increased the heterogeneity of the meta-analysis that Yang et al. only analyze PAS.

Our second concern is that Yang et al. incorrectly use standard error (SE) instead of standard deviation (SD). We note that Khedr et al. (2009) and Khedr and Abo-Elfetoh (2010) reported SE, but Yang et al. did not appear to convert SE to SD before the meta-analysis. Means of control and experimental groups also appeared to be swapped in some studies. For data extraction, Yang et al. did not define the time points of extracted outcome data, and the simulated data came from several different follow-up times. Yang et al. stated that “We calculated the mean scores (Mean) and standards Deviations (SD) before and after interventions according to the calculated inequality of the equator to guidance in the Cochrane Handbook for Systematic Reviews of Intervention,” but data extracted from a study (Kim et al., 2011) belonged to change values.

We added a randomized controlled trial (Du et al., 2016) to the present meta-analysis and extracted data according to the following criteria: (1) assessing after intervention immediately; (2) re-extraction of DD and PAS values in the form of Mean and SD; (3) uniforming the type of liquids to measure PAS values (patients with dysphagia have more difficulty in swallowing liquids than semi-solids and liquid is an important cause of aspiration pneumonia; Winstein et al., 2016). We converted SE to SD for each group of study and analyzed all of the data according to the recommendations of the Cochrane manual.

As shown in Figure 1, we reproduced the meta-analysis using revman5.3 with the correct data, which led to a greater effect size [SMD = −0.87 (−1.22, −0.52), *p* < 0.001] than Yang et al. [SMD = 0.65 (0.04, 1.26), *p* = 0.04]. Heterogeneity also greatly reduced [tau^2^ = 0.15, *I*^2^ = 41%] compared with Yang et al. [tau^2^ = 0.67, *I*^2^ = 74%]. Subgroup analysis was conducted according to different frequencies. The efficacy of high-frequency stimulation [SMD = −0.82 (−1.28, 0.36), *p* = 0.0005] and low-frequency stimulation [SMD = −0.97 (−1.56, −0.38), *p* = 0.001] in the treatment of dysphagia after stroke was greater than conventional rehabilitation, which was completely different from Yang's results. The results showed that rTMS was superior to conventional rehabilitation in the treatment of post-stroke dysphagia regardless of stimulation frequency. We emphasize the importance of carefully examining the data extraction and reviewing the results of the meta-analysis.

**Figure 1:**
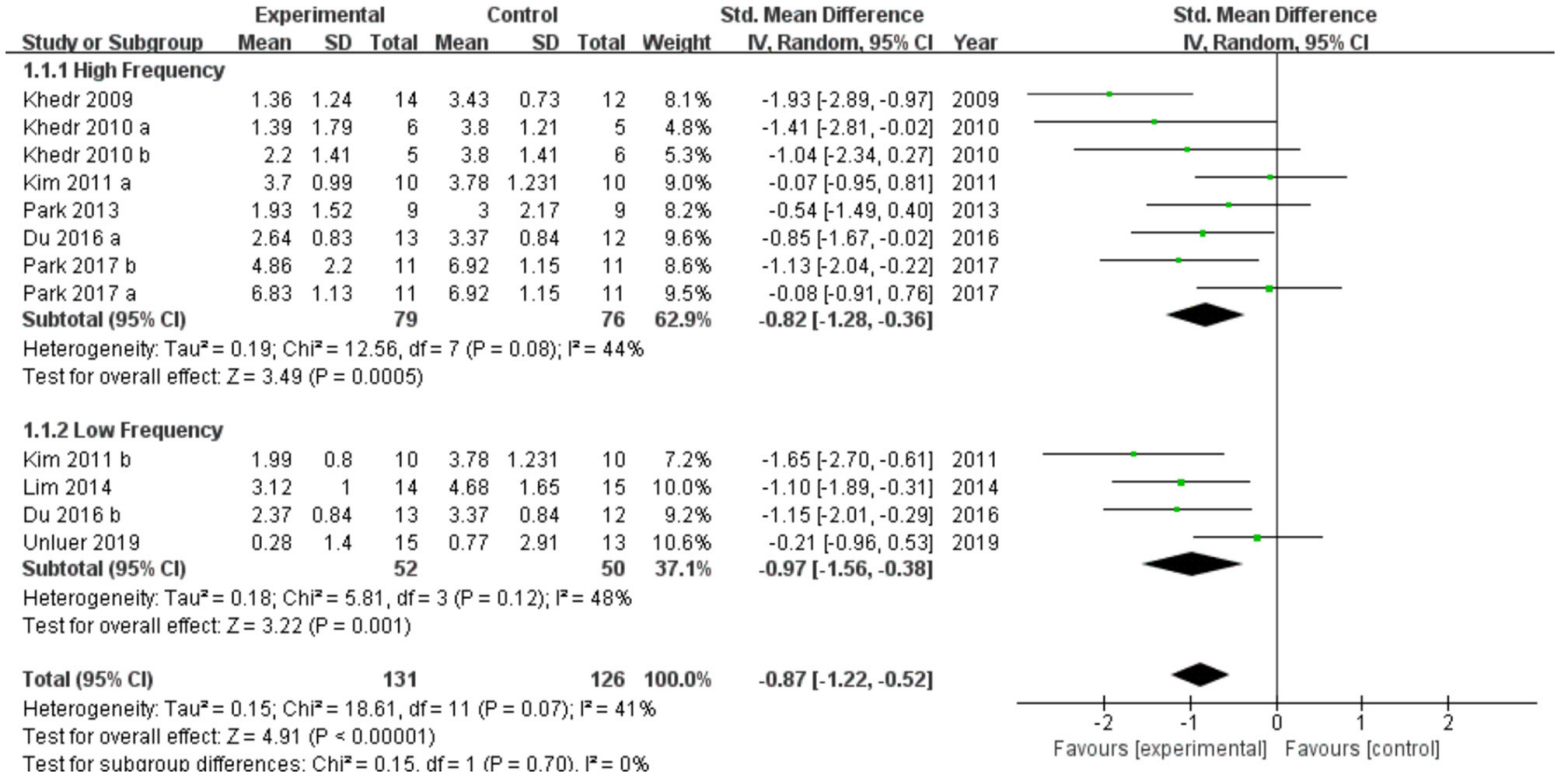
Forest plot of changes from baseline to checkpoint for swallowing function. SMD, Standardized mean difference; CI, confidence interval; N, number of participants.

In conclusion, we urge readers to be cautious about the quality and validity of the review analyzed by Yang et al. The study omitted several important trials and produced extraction/synthesis errors in the meta-analysis, leading the authors to underestimate the efficacy of rTMS in patients with post-stroke dysphagia.

## Author Contributions

Y-lX, SW, and Y-hX conceived the idea and wrote the manuscript. QW and YW analyzed the data. All authors reviewed the final manuscript.

## Funding

YW was supported by Sichuan Medical Research Project Plan (Q18038). QW was supported by Development Project of Affiliated Hospital of North Sichuan Medical College (2021ZD014). Y-lX was supported by the North Sichuan Medical College of Medical Sciences Postgraduate Research Scholarship.

## Conflict of Interest

The authors declare that the research was conducted in the absence of any commercial or financial relationships that could be construed as a potential conflict of interest.

## Publisher's Note

All claims expressed in this article are solely those of the authors and do not necessarily represent those of their affiliated organizations, or those of the publisher, the editors and the reviewers. Any product that may be evaluated in this article, or claim that may be made by its manufacturer, is not guaranteed or endorsed by the publisher.
